# Undiagnosed Wilson’s Disease and Fibromyalgia Masking Bowel Perforation

**DOI:** 10.7759/cureus.13504

**Published:** 2021-02-23

**Authors:** Tyler Culpepper, Amar H Kelkar

**Affiliations:** 1 Medicine, University of Florida College of Medicine, Gainesville, USA

**Keywords:** delayed diagnosis, ceruloplasmin, bipolar disorder, wilson’s disease, psychiatric disease, leipzig score, laxative abuse, high-value care, cathartic abuse, elevated transaminases

## Abstract

Wilson’s disease is a complex multi-organ disease characterized by impaired biliary excretion of copper and resulting deposition of excess copper in the liver and other organs. It has a wide range of clinical presentations, and diagnosis often requires a high degree of clinical suspicion, especially in patients with multiple other comorbidities.

We present the case of a 37-year-old woman with a complex medical and psychiatric history who was admitted for chronic diarrhea, hepatic enzyme elevation, electrolyte abnormalities, hyperammonemia, and methicillin-sensitive *Staphylococcus aureus* bacteremia. She was eventually found to have low serum ceruloplasmin level and elevated urine copper levels. Though confirmatory liver biopsy was not performed due to bowel wall rupture and septic shock, most of her symptoms and lab abnormalities could be explained by an underlying diagnosis of Wilson’s disease.

We present this case primarily as a cautionary tale. This patient was not lacking in medical attention prior to this prolonged hospitalization; however, her psychiatric issues and fibromyalgia management were the predominant foci during her frequent primary care office visits and likely distracted from the patient’s chronic laboratory abnormalities. More vigilant laboratory evaluation of underlying medical conditions in psychiatric patients may be warranted in order to prevent serious complications of such conditions.

## Introduction

A 37-year-old woman presented to our facility for management of persistent methicillin-sensitive Staphylococcus aureus (MSSA) bacteremia and worsening confusion. The patient had a complex medical and psychiatric history and a broad range of reported symptoms. Management of the case was convoluted, involving diagnoses of acute medical issues and their interactions with known and previously unknown underlying medical conditions. Despite close observation and a meticulous approach, the patient showed only mild improvement, and subsequently declined rapidly following exploratory surgery.

## Case presentation

A 37-year-old woman presented with confusion resulting in a fall down the stairs at her home. The patient was initially admitted at an outlying facility for acute mental status change and was transferred to our facility for management of persistent MSSA bacteremia and worsening confusion. Her past medical history included fibromyalgia, microscopic colitis with chronic secretory diarrhea, severe chronic obstructive pulmonary disease (COPD), and recent intentional weight loss of 100-pounds. The patient had a psychiatric history of borderline personality disorder and bipolar I disorder without history of psychotic features. She had a remote history of alcohol abuse and had quit drinking more than 15 years prior to admission.

At the outside hospital, on presentation her vitals were remarkable for heart rate 138 beats per minute and hypothermia (T = 35.0C). Blood pressure, respiratory rate, and oxygen saturation were normal. Physical exam revealed an awake, alert, lethargic female with dry mucous membranes; normal heart, lung and bowel sounds; no abdominal masses, tenderness, or organomegaly; no jugular venous distension (JVD) or peripheral edema; no focal neurological deficits (including cranial nerves) and normal reflexes. Serum labs revealed a sodium level of 136 mmol/dL, potassium level of 2.5 mmol/dL, sodium bicarbonate level of 16 mmol/dL with an anion gap of 16 mmol/L, magnesium level of 1.2 mg/dL, creatinine level of 1.26 mg/dL, blood urea nitrogen (BUN) level of 45 mg/dL, aspartate transaminase (AST) of 62 U/L, alanine transaminase (ALT) of 60 U/L, alkaline phosphatase (ALP) of 130 U/L, total bilirubin level of 0.4 mg/dL, albumin level of 2.5 g/dL, and ammonia of 174 umol/L. pH was 7.27 on an arterial blood gas. Inflammatory markers were elevated with an erythrocyte sedimentation rate equal to 48 mm/hour and C-reactive protein greater than 160 mg/L. A complete blood count showed white blood cells equal to 10.4 cells/uL, and a hemoglobin and platelet level of 12.3 g/dL and 213,000/mm^3^, respectively. Thyroid function and urinalysis was normal but a urine drug screen revealed benzodiazepines and opiates. Her pain and anxiolytic medications were held, she was started on empiric broad spectrum antibiotics, and blood cultures later revealed methicillin sensitive Staphylococcus aureus (MSSA) in one out of two bottles with repeat culture revealing this in two out of two bottles. She was transferred to our facility on hospital day 4 for further evaluation of encephalopathy and management of persistent MSSA bacteremia. The total duration of hospitalization was two months.

On arrival to our facility, she was found to be somnolent but arousable by verbal stimuli, had visual agnosia, and had diffuse abdominal tenderness without rebound tenderness or guarding. Due to chronic diarrhea with electrolyte abnormalities, the patient had a Mediport™ in place. Her laboratory evaluation showed severe non-anion gap metabolic acidosis with hypokalemia (potassium level of 2.7 mg/dL) and hypomagnesemia (magnesium level of 1.2 mg/dL). She had 25-hydroxy-vitamin D inadequacy with a level of 18 ng/mL. She was found to have an elevated ammonia level of 99 μmol/L, elevated international normalized ratio (INR) of 1.7, thrombocytopenia of 92,000/mm^3^, hyperbilirubinemia with a level of 2.5 μmol/L, and AST/ALT (119/102 U/L) values consistent with hepatocellular injury.

**Figure 1:**
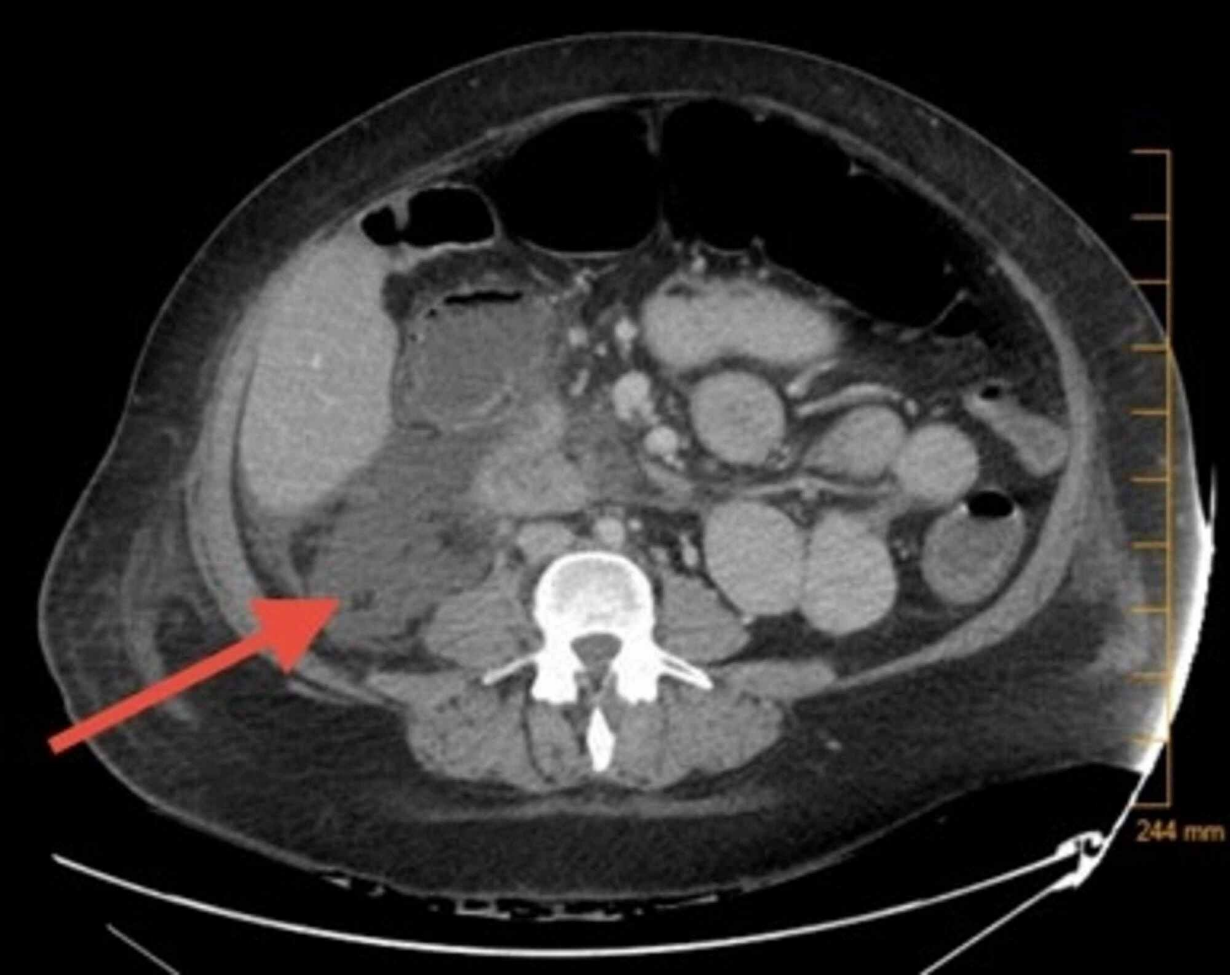
Abdominal CT scan demonstrating a right perinephric fluid collection as indicated by the arrow CT: Computed tomography

Given these findings, the patient underwent chronic liver disease workup which revealed low serum ceruloplasmin levels of 19 mg/dL (normal range: 20-60 mg/dL) that was suspected to be falsely elevated due to ceruloplasmin being an acute-phase reactant. Subsequently, 24-hour urine copper was found to be elevated at 133 mcg/spec (normal: 15-60 mcg/spec). Antinuclear antibodies, anti-smooth muscle antibody, and anti-liver/kidney microsome type 1 antibody were negative. An acute viral hepatitis panel was negative. Her MELD score was calculated as 12 with a three-month estimated mortality of 6% and Child-Pugh score was 9 with Child Class B. Computed tomography (CT) of the abdomen demonstrated a fatty liver and a right perinephric fluid collection concerning for a urinoma versus an infectious process (Figure 1). Ophthalmologic evaluation did not note any Kayser-Fleischer (KF) rings. A liver biopsy could not be attained due to ongoing bacteremia.

One week prior to this admission, she was admitted for chronic diarrhea and esophagogastroduodenoscopy showed changes consistent with chronic gastritis. Endoscopic retrograde cholangiopancreatography, performed due to concerns obstructive pathology in the setting of elevated bilirubin, with duodenal biopsies showed signs of chronic inflammation but no evidence of celiac disease. A prior colonoscopy had shown microscopic colitis, but a repeat colonoscopy showed chronic nonspecific inflammation. At the time of transfer to our facility, several empty bottles of bisacodyl laxatives were found in her bag by the nursing staff, raising concerns for laxative abuse.

Over the course of the admission, MSSA bacteremia was treated with intravenous nafcillin infusions, but blood cultures remained persistently positive. Her Mediport™ was removed due to concerns for a central line-associated bloodstream infection. She underwent exploratory laparoscopy due to her intraabdominal fluid collection which was urgently converted to an open laparotomy when she was found to have severe bowel necrosis and perforation of the ascending and transverse colon with stool and pus collections in the peritoneum. After significant bowel resection, the patient had a prolonged stay in the surgical intensive care unit. She was discharged with recommendations to obtain a liver biopsy or ATP7B genetic testing and initiation of Trientine. Later testing for ATP7B mutations confirmed Wilson's disease; however, she did not undergo liver biopsy or elastography. Three months after hospital discharge, chart review revealed she was still being actively managed by psychiatry. Thus, her symptoms had not resolved with Trientine.

## Discussion

The patient’s presentation was initially most concerning for sepsis, as evidenced by both previously used systemic inflammatory response syndrome (SIRS) criteria (tachycardia and hypotension) and the latest definition including organ dysfunction (altered mental status and serum total bilirubin = 1.4) with her port initially as a suspected source, later proven with bacteremia and retroperitoneal abscess found on imaging [1]. Her sepsis was likely the latest sequela of her Wilson disease (WD), as it was thought to be secondary to bowel perforation as a result of non-perforating trauma versus her prior colonoscopy in the setting of excessive surreptitious laxative use (as evidenced by her electrolyte derangements, excessive weight loss, and the discovery of empty laxative bottles) which was thought to be secondary to her underlying psychiatric conditions, which we now know were likely secondary to Wilson’s disease. However, with recent initiatives focused on identifying and treating sepsis early, this was likely at the forefront of the clinical thought process on admission and was confounded by the presence of a port [2].

This was a particularly challenging clinical scenario. The patient presented with a plethora of symptoms and findings, confounding her final diagnoses. Though many of the signs and symptoms pointed towards WD, there were other plausible alternative explanations. Interestingly, during the initial workup, the patient was also evaluated for liver disease. Initially, alpha-1-antitrypsin deficiency was suspected due to the early development of COPD with only a mild smoking history; however, laboratory studies revealed elevated alpha-1-antitrypsin levels and low ceruloplasmin.

While the patient was treated appropriately upon admission with regard to the one of the most acute and life-threatening conditions (i.e., sepsis), she met criteria for acute liver failure (encephalopathy with INR >1.5), which was not immediately addressed, likely due to both the variability in the definition of acute liver failure and the absence of the remarkable transaminase abnormalities seen in acute liver failure of other etiologies [3-4]. This may have been why the diagnosis of her underlying WD was missed on admission. Furthermore, she would likely have benefitted from a more extensive outpatient workup for her underlying psychiatric conditions, as this type of evaluation may have prevented her missed diagnosis of WD as an outpatient, her admission described herein, and the underlying factors that led to it. We will analyze each of the presenting symptoms and laboratory abnormalities separately in our attempt to clarify this delayed diagnosis of Wilson’s disease.

WD has a wide range of clinical presentations besides chronic liver dysfunction. The extrahepatic clinical presentations include neuropsychiatric disturbances, renal tubular acidosis (RTA), Fanconi syndrome, Coombs-negative hemolytic anemia, abnormalities of the bone, dystonia, tremors, dysautonomia, migraine headaches, insomnia, depression, anxiety, and frank psychosis [5-6]. Younger patients present more often with acute liver failure, while older patients can present with progressive neurological disorders [5]. In the 8th International Meeting on Wilson disease and Menkes disease at Leipzig, a diagnostic scoring system for WD was proposed and the scoring included clinical and biochemical features [7]. According to Leipzig scoring system, scores of 0 to 1 made diagnosis unlikely, scores of 2 to 3 made diagnosis probable, but requiring further testing, and scores of 4 and higher made diagnosis highly likely. While evaluating our patient, the Leipzig scoring system was a useful clinical tool, especially given the atypical presentation of WD. Our patient’s Leipzig score was 4. Additionally, the American Association for the Study of Liver Diseases has an algorithm for the diagnosis of WD, and for this patient, liver biopsy was the next recommended step to confirm the diagnosis [5].

Within the liver, copper is normally incorporated into apoceruloplasmin to form holo-ceruloplasmin, commonly known as ceruloplasmin. In WD, the most common mutations have been found in the ATP7B gene on chromosome 12; however, genetic diagnosis is often limited due to limited access to genetic analysis and multiple possible associated gene mutations. ATP7B mutations affect the transport of copper from the cellular periphery to the Golgi apparatus where it normally becomes bound to apoceruloplasmin. The failure of this process causes retention of copper within the liver [8]. As a result, hepatic production of ceruloplasmin is reduced. When combined with the short half-life of apoceruloplasmin, this results in low serum levels of ceruloplasmin. Copper overload leads to hepatocellular injury, largely from free radical damage, resulting in both apoptosis and necrosis, release of copper ions into the serum, and heavy loss of copper in urine [5]. In this case, the patient had a low serum ceruloplasmin level and significantly elevated 24-hour urine copper excretion, consistent with diagnostic criteria for WD.

The patient’s electrolyte deficiencies were also a point of uncertainty throughout the case. The patient had persistent hypokalemia, hypomagnesemia, hyperchloremia, and low bicarbonate levels without significant metabolic acidosis. The metabolic derangements seen in this patient were initially thought to be due to her chronic diarrhea from microscopic colitis. On a closer look, another possible cause of her metabolic abnormalities emerged. Hyperchloremia with low plasma bicarbonate and hypokalemia can be seen in RTA, both proximal and distal, in the context of diarrhea and chronic respiratory alkalosis. Our patient had non-anion gap metabolic acidosis with arterial blood gas analysis showing a pH of 7.27, pCO2 of 18.4 mmHg, and bicarbonate level of 39 mg/dL. Urine anion gap was positive at 36 and urine pH was 6. Her serum calcium levels had been chronically low between 7-8 mg/dL. A positive urine anion gap would not be expected if diarrhea were causing the metabolic acidosis and urine pH would be lower than 5.5 in proximal RTA. Thus, the weight of evidence leaned towards type I (distal) RTA. WD has been known to cause RTA, which might have been the case in our patient [9].

The patient had low 25-hydroxy-vitamin D levels of 18 ng/dL on admission. WD can cause low vitamin D levels via two mechanisms. Loss of bicarbonate ions in the urine causes bone demineralization with loss of bicarbonate and eventual loss of calcium. Subsequent metabolic acidosis inhibits hydroxylation of 25(OH) vitamin D3 to 1, 25(OH) vitamin D3, which in turn leads to diminished calcium absorption from the intestine. Chronic bone demineralization would eventually lead to hypocalcemia [9]. On chart review, our patient had chronic hypocalcemia. Underlying osteoporosis could have been a contributing factor to her chronic arthralgias and myalgias, which were considerations in her prior diagnosis of fibromyalgia.

The patient had also presented with hyperammonemia that worsened during the first few days of admission and was later controlled with lactulose. Given the evidence of hepatocellular injury, coagulopathy, low ceruloplasmin, elevated urine copper, the presence of hyperammonemia was considered to be secondary to true liver disease. Non-hepatic causes of hyperammonemia including medications, infection in a neurogenic bladder, and ureterosigmoidoscopy were considered unlikely, given the history and presentation of the case [10]. Abdominal imaging showed diffusely increased echogenicity of the liver parenchyma, consistent with hepatic steatosis without notable nodularity. Due to the complex and prolonged hospital course, as well as bacteremia and peritonitis, a liver biopsy could not be obtained. Thus, liver fibrosis could not be ruled out and copper overload could not be confirmed.

The patient had a prior diagnosis of bipolar disorder. Neuropsychiatric presentation of WD includes depression, anxiety, personality changes and bipolar disorder [6]. It was suspected that bipolar disorder was an earlier manifestation of the underlying WD. On brain imaging with CT head and magnetic resonance imaging, our patient had no structural abnormalities in the basal ganglia.

Our patient did not have KF rings on ophthalmological examination. KF rings are seen in 44-62% of WD patients presenting with mainly hepatic disease [5]. There was also no mention of sunflower cataracts on her ophthalmological examination.

We present this case primarily as a cautionary tale. This patient was not lacking in medical attention, being seen by a primary care physician approximately four times yearly; however, her psychiatric issues and fibromyalgia management dominated her office visits, and likely distracted from the patient’s other medical needs. This delay in diagnosis should serve as a reminder to healthcare professionals about the various presenting features of WD and that psychiatric symptoms may precede the recognition of hepatic involvement [6].

Unfortunately, routine screening of psychiatric patients for WD is not high yield, and WD is often not present in these patients despite low serum ceruloplasmin concentrations [11]. Therefore, further confirmatory testing (e.g., urinary copper concentrations and perhaps genetic analyses) is often required to arrive at the diagnosis, and such testing is a challenge in a practice culture aimed to provide high-value care. This and similar cases may provide justification for more expansive laboratory testing in patients with psychiatric conditions, and may, in fact, increase high-value care if it leads to fewer hospital admissions, surgeries, and prolonged ICU courses, as was the case for this patient.

## Conclusions

WD is a complex condition with multiorgan involvement that can present with various constellations of symptoms. Because a definitive diagnosis of WD requires either an invasive biopsy or a cumbersome urine collection, an understanding of the full spectrum of clinical presentation of WD and a high degree of clinical suspicion is required for the diagnosis, especially in cases where the studies required for definitive diagnosis are unsafe or impossible to obtain. This case demonstrates an example of psychiatric, infectious and gastrointestinal complications that commanded the attention of the patient’s physicians, distracting them from the patient’s chronic mild underlying laboratory abnormalities suggestive of a hepatic process. Early diagnosis could lead to prompt initiation of treatment and halt progression of disease and development of complications such as those seen in this case.
